# Local and Systemic Reaction after Receiving Coronavirus Disease Vaccines among Healthcare Workers in Sapporo, Japan

**DOI:** 10.31662/jmaj.2021-0103

**Published:** 2021-09-13

**Authors:** Yoshinosuke Shimamura, Yoshiyasu Anbo, Yasushi Furuta

**Affiliations:** 1 Office of Infection Control and Prevention, Teine Keijinkai Medical Center, Sapporo, Japan

**Keywords:** COVID-19 vaccine, healthcare workers, Japan, postvaccination reactions

## Introduction

Japan started coronavirus disease 2019 (COVID-19) vaccine administration for healthcare workers (HCWs) in February 2021^(1)^. The inoculations were gradually expanded to the elderly population of those 65 years old or older, followed by those with underlying health conditions and finally the general public^(2)^. Although it is crucial for both healthcare professionals and the general public to share information about postvaccination reactions, the reports of COVID-19 vaccination experience outside of clinical trial settings were still limited^(3)^. Therefore, we report local and systemic reactions after each dose of mRNA-based COVID-19 vaccines manufactured by the Pfizer-BioNTech among HCWs at the Teine Keijinkai Medical Center, a 670-bed tertiary medical center in Sapporo, Hokkaido, Japan.

## Methods

From March 12, 2021, to April 9, 2021, a total of 1990 HCWs were scheduled to receive the Pfizer-BioNTech vaccine, according to the manufacturer’s instructions. Data were collected using Microsoft Forms (Microsoft, WA, USA). HCWs received smartphone text messages and weblink to initiate a Web-based questionnaire two consecutive days after each vaccine administration, and they were asked questions about local reactions (pain, redness, and swelling)^(4)^, systemic reactions (headache, fatigue, myalgia, arthralgia, nausea/vomiting, chills, and fever ≥ 38℃)^(4)^, and anaphylaxis reaction^(5)^. Information about those who took antipyretics and analgesics, including acetaminophen and nonsteroidal anti-inflammatory drugs, was also collected. We summarized postvaccination reactions as numbers and percentages. The participants in this report provided their written informed consent for data collection and publication. This study was conducted in compliance with the Declaration of Helsinki and was approved by the institutional review board of Teine Keijinkai Medical Center (approval number 2-020260-00).

## Results

As presented in Table 1, a total of 1628 (82%) and 1739 (87%) persons responded to at least one online questionnaire within 2 days following the first and second vaccine dose, respectively. The median age was 32 years (4.0% were 60 years old or older), and 21% were men. The common reported reactions after the first dose of vaccine were injected site pain (92.5% at day 1; 67.2% at day 2), fatigue (17.3% at day 1; 12.9% at day 2), injection site swelling (11.2% at day 1; 5.2% at day 2), and headache (9.6% at day 1; 8.2% at day 2). Anaphylaxis reaction was developed in two HCWs (0.1%) after the first dose, both of which were to be exempt from the second dose; one had a history of bronchial asthma treated with inhaled corticosteroids, and another one had a history of erythema exudative multiforme against nonsteroidal anti-inflammatory drugs. As a result, the remaining 1988 HCWs, except for 2 HCWs who had developed anaphylaxis after the first dose, received the second vaccine dose. Systemic and local reactions were greater after the second dose, including injected site pain (94.8% at day 1; 79.5% at day 2), fatigue (45.9% at day 1; 43.4% at day 2), headache (29.4% at day 1; 29.8% at day 2), chills (19.6% at day 1; 19.2% at day 2), and fever ≥ 38℃ (7.5% at day 1; 9.1% at day 2). Anaphylaxis reaction was developed in two different HCWs (0.1%) after the second dose. Of note, a total of 180 HCWs were absent from work or left work early following the vaccine administration. Additionally, 944 HCWs (47.5%) reported taking antipyretics and analgesics following vaccine administration before the appearance of postvaccination reactions.

**Table 1.** Local and Systemic Reactions to mRNA COVID-19 Vaccines Reported 1-2 Days after Vaccination.

	Dose 1: day 1 （n = 1628）	Dose 1: day 2 （n = 1647）	Dose 2: day 1 （n = 1784）	Dose 2: day 2 （n = 1739）
Local pain, n (%)	1506 (92.5)	1094 (67.2)	1691 (94.8)	1383 (79.5)
Redness, n (%)	47 (2.9)	26 (1.6)	43 (2.4)	55 (3.2)
Swelling, n (%)	182 (11.2)	85 (5.2)	280 (15.7)	216 (12.4)
Headache, n (%)	157 (9.6)	134 (8.2)	525 (29.4)	519 (29.8)
Fatigue, n (%)	282 (17.3)	210 (12.9)	819 (45.9)	755 (43.4)
Myalgia, n (%)	56 (3.4)	49 (3.0)	223 (12.5)	228 (13.1)
Arthralgia, n (%)	24 (1.5)	44 (2.7)	263 (14.7)	274 (15.8)
Nausea/vomiting, n (%)	28 (1.7)	19 (1.2)	109 (6.1)	77 (4.4)
Chills, n (%)	46 (2.8)	29 (1.8)	349 (19.6)	334 (19.2)
Fever ≥ 38℃, n (%)	3 (0.2)	10 (0.6)	134 (7.5)	159 (9.1)

## Discussion

Our study demonstrated that postvaccination reactions, especially systemic reactions, were more frequently reported after the second dose. Additionally, most of these reactions reached their peaks 1 day after the vaccine administration, except for myalgia, arthralgia, and fever. Our results were generally compatible with existing literature; however, the frequency of fatigue, headache, and fever ≥ 38℃ of this cohort was lower than that in previous reports^(3), (6), (7), (8)^. This discrepancy may be related to differences in the sample size and study population because our cohort had a smaller sample size and smaller proportion of persons 60 years or older^(6), (7), (8)^. This may also be explained by the fact that 47.5% of the HCWs had taken antipyretics and analgesics following the vaccine administration even before the appearance of postvaccination reactions, contributing to reduced frequency of systemic postvaccination reactions. Moreover, there is a possibility that postvaccination reactions could have been underreported because the participation in smartphone-based questionnaire system was voluntary and approximately 10% of the HCWs were not responded to the questionnaire. Additionally, we found that a total of 180 vaccinated HCWs were absent from work or left work early after the vaccine, which may suggest that work shift coverage and special leave of absence should be considered in hospital-wide vaccine administrations.

The generalizability of our findings to other populations may be limited because our study population was limited to HCWs at a Japanese tertiary care institution. Nonetheless, our findings may contribute to the ongoing nationwide vaccine campaign to control the spread of COVID-19 by providing information about postvaccination reactions in the real-world setting. Finally, the present results have several implications. First, clinicians need to inform vaccine recipients that postvaccination reactions are common and may affect their ability to do daily activities. Second, clinicians should reassure the recipients that most of these reactions are transient and resolve in several days. More importantly, we, as clinicians, encourage the public to receive the COVID-19 vaccines to decrease the further loss of life resulting from the global COVID-19 pandemic.

## Article Information

### Conflicts of Interest

None

### Acknowledgement

We thank all members of the office of infection control and prevention at Teine Keijinkai Medical Center.

### Author Contributions

Conceptualization, YS, YA, and YF; data curation, YS, YA, and YF; formal analysis, YS, YA, and YF; investigation, YS, YA, and YF; methodology, YS, YA, and YF; project administration, YF; resources, YS, YA, and YF; supervision, YA and YF; visualization, YS, YA, and YF; writing - original draft, YS, YA, and YF; writing - review and editing, YS, YA, and YF.

### Approval by Institutional Review Board (IRB)

This report was conducted in compliance with the Declaration of Helsinki and was approved by the institutional review board of Teine Keijinkai Medical Center (approval number 2-020260-00).
